# Ischaemia in tuberculous meningitis: Update on pathophysiology, clinical predictors and management

**DOI:** 10.1097/QCO.0000000000001194

**Published:** 2026-04-08

**Authors:** Georgia Lamb, Reinout van Crevel, Graeme Meintjes, Angharad Davis

**Affiliations:** 1 Department of Infectious Diseases, Barts Health NHS Trust, The Royal London Hospital, Whitechapel Rd, London E1 1FR; 2 Queen Mary and Barts Health TB Centre, Centre for Immunobiology and Infection, Blizard Institute, Queen Mary University London, 4 Newark St, London E1 2AT; 3 Department of Internal Medicine and Radboud Community for Infectious Diseases, Radboud University Medical Center, Nijmegen, the Netherlands; 4 Wellcome Discovery Research Platforms in Infection, Centre for Infectious Diseases Research in Africa, Institute of Infectious Disease and Molecular Medicine, University of Cape Town, Observatory 7925, Republic of South Africa; 5 Department of Medicine, University of Cape Town, Observatory 7925, Republic of South Africa; 6 Francis Crick Institute, London, NW1 1AT, UK

**Keywords:** Tuberculous meningitis, stroke, neuroinflammation, host-directed therapy, intracranial pressure

## Abstract

**Purpose of review:**

Tuberculous meningitis (TBM) is a severe manifestation of *Mycobacterium tuberculosis* infection, associated with high mortality and long-term neurological disability. Cerebral ischaemia and infarction are major contributors to poor outcomes, yet the underlying mechanisms remain incompletely understood. This review summarises current understanding of the pathophysiology, predictors, and emerging therapies for TBM-associated ischaemia, highlighting critical research priorities.

**Recent findings:**

Stroke in TBM reflects a complex interplay of neuroinflammation, immunothrombosis, and raised intracranial pressure (ICP). Key emerging mediators include neutrophil extracellular traps, matrix metalloproteinases, pro-inflammatory cytokines (TNF-α, IL-1β, IL-6), platelet hyperactivation, and dysregulated tryptophan metabolism. Advanced neuroimaging, particularly vessel wall imaging, may improve infarction risk prediction and patient stratification, potentially supported by machine learning approaches. Large trials of adjunctive antiplatelet therapy show limited or inconsistent benefit, while small studies suggest anti-TNF therapy may be beneficial.

**Summary:**

Despite progress in characterizing inflammation, thrombosis, and vascular injury in TBM, significant gaps remain in understanding mechanisms and timing of stroke. Improved mechanistic insight, integrated translational research, and trials of novel host-directed therapies are needed to prevent stroke and improve neurological outcomes. In parallel, efforts should focus on optimising existing strategies, particularly defining effective approaches to management of raised ICP.

## Introduction

Tuberculous meningitis (TBM) is the most severe manifestation of *Mycobacterium tuberculosis (M.tb)* infection, associated with high mortality and long-term neurological morbidity. An estimated 10.7 million cases of tuberculosis occurred in 2024 [1]; TBM accounts for 1.3-6.8% of all tuberculosis cases, whilst the prevalence of TBM amongst people living with HIV reaches 13.6% [2,3]. Adult mortality ranges from 27-48%, with higher case-fatality rates in children and HIV co-infection, and 30-50% of survivors experience long-term neurological sequelae [2–5].

Cerebral ischaemia and infarction (stroke) are among the most devastating TBM complications, occurring in 15-57% of patients and conferring a threefold increase in mortality [5, 6]. Despite this burden, the mechanisms underlying TBM-associated ischaemia remain incompletely defined, and effective preventive strategies are lacking. Early identification of patients at high risk of stroke and development of targeted host-directed therapies represent major unmet needs.

TBM pathology is largely immune-mediated, prompting interest in adjuvant host-directed therapies including antiplatelet and immunomodulatory agents. However, evidence supporting these interventions for managing TBM-associated stroke is inconsistent or lacking [8]. International guidelines conclude there is insufficient evidence to recommend aspirin, thalidomide, or infliximab as adjunctive therapies in TBM [9]. Corticosteroids are the only host-directed therapy proven to reduce mortality, yet they do not reduce stroke incidence [10].

In this review, we synthesise current evidence on the pathophysiology of TBM-associated ischaemia from clinical, neuroimaging, and *ex vivo* studies, examine targeted therapeutic strategies, and highlight priorities for future research.

### Part 1: Pathophysiology of inflammation and ischaemia in TBM

Ischaemia in TBM is driven by several pathogenic processes. Basal inflammatory exudate can surround and inflame arterial walls, causing large and perforator vessel thrombotic occlusion, while diffuse inflammatory vasculopathy causes luminal narrowing and reduced cerebral perfusion [5, 7]. Despite advances in characterising TBM neuroinflammation, mechanisms linking inflammation to cerebrovascular injury remain incompletely defined. Here, we review recent evidence for pathways connecting inflammation and ischaemia.

#### Vascular endothelial-derived growth factor (VEGF)

VEGF is released from endothelial cells in response to hypoxia and regulates endovascular permeability. In cerebral ischaemia, VEGF has protective and pathogenic roles, promoting angiogenesis but also blood-brain barrier (BBB) dysfunction, which implicates it in early TBM pathology [11]. A 2025 study found higher CSF VEGF levels in 222 TBM patients compared to controls, and VEGF predicted mortality, although this only reached statsitical signicifance in a discovery cohort and not in the validation cohort[12*]. A prior smaller study reported no overall increase in CSF or serum VEGF in TBM; whilst serum VEGF correlated with basal exudates on MRI, it was not independently associated with stroke [13*].

Intra-vitreal anti-VEGF therapy bevacuzimab improves outcomes in ocular TB and TB-immune reconstitution inflammatory syndrome (IRIS), where VEGF-driven angiogenesis promotes vasculitis and endovascular leakage [14, 15]. Experimental models suggest complex roles for VEGF signalling: IQGAP1, a scaffolding protein upregulated in *Mycobacteria marinum*-infected macrophages, promotes VEGF production, granuloma formation, and mycobacterial survival whilst suppressing TNF signalling. Pharmacological inhibition of IQGAP1 reduced VEGF production and tissue damage in *ex-vivo* models, identifying a potential therapeutic target [16*]. Although VEGF is linked to neuroinflammation and mortality in TBM, its role in TBM-associated infarction remains uncharacterised.

#### Neutrophils, Neutrophil extracellular traps and Matrix metalloproteinases

Neutrophils are central to innate immunity in TBM. Phagocytosis of *M.tb-*infected neutrophils and subsequent necrosis drive tissue damage, while peripheral neutrophils secrete pro-inflammatory cytokines including TNF-α and interleukin (IL)-1β [11]. Neutrophil extracellular traps (NETs) are web-like structures released by activated neutrophils, designed to capture and kill bacteria. NETs amplify macrophage activation and cytokine release (IL-6, TNF, IL-1β, IL-10), [17] and enhance thrombosis by activating platelets, inhibiting fibrinolysis, and providing a scaffold for clot formation. Activated platelets further drive NET formation, creating a self-reinforcing immunothrombotic cycle [18]. Higher pre-treatment CSF neutrophil counts correlate with mortality and stroke [19, 20]; this likely reflects disease severity, and whether neutrophil-driven thrombosis is causal remains unclear.

Matrix metalloproteinases (MMPs), secreted by astrocytes, microglia and neutrophils, degrade extracellular matrix and BBB components, facilitating leukocyte recruitment and vascular injury [11]. *In vitro*, astrocytes and microglia exposed to *M.tb*-infected monocytes secrete MMP-1, -3 and -9, with downregulated MMP-2, driven by pro-inflammatory cytokines including TNF-α [21]. In a cohort of 222 adult TBM patients, elevated CSF MMP-10 predicted mortality and genetic traits linked to CSF MMP-10 levels predicted mortality in a second group of 218 TBM patients, supporting a causal role for MMP-10 [12*].

Paediatric TBM studies are heterogenous. Rohlwink et al. demonstrated elevated CSF MMP-2 and -9 correlated with severity and neurological complications [22], while Manyelo et al. observed reduced CSF MMP-1 in children with stroke [23]. Transcriptomic analysis showed higher systemic MMP-8 expression in children with cerebral infarction compared to those without, with no differences in TNF-α, IL-1β, IL-10, IFN-γ, or VEGF [24*]. Poh et al identified marked CSF upregulation of MMP-1, -3, -7, -9 and -10 and NETs in TBM compared with controls, associated with leptomeningeal enhancement on neuroimaging, and high CSF MMP-7 and -10 predicted death or disability [25**]. In a murine TBM model, addition of doxycycline (an MMP inhibitor) to anti-tuberculosis therapy (ATT) improved survival, reduced CSF bacillary load and preserved intracranial vasculature, supporting MMP inhibition as a therapeutic strategy.

#### Platelet activation and thrombosis

*M.tb* infection induces clinical thrombocytosis, and plasma markers of platelet activation are upregulated in patients with TB [26, 27]. Activated platelets interact with neutrophils and monocytes to promote NET formation and MMP expression, promoting a pro-inflammatory, matrix-degrading phenotype [27, 28]. Transcriptomic analyses identified an upregulated haemostasis and platelet activation gene module correlating with disease severity, but not mortality, alongside marked neutrophil activation [29*].

Indices of platelet activation and heterogeneity (mean platelet volume, platelet distribution width, platelet-large cell ratio, platelet aggregometry) are associated with cerebral infarction in TBM, whereas conventional coagulation parameters (INR, clotting time) are not [30*]. These data support immunothrombosis (aberrant clot formation driven by innate immune responses) rather than classical hypercoagulability as the major driver of stroke.

#### Pro-inflammatory cytokines

Pro-inflammatory cytokines are essential for mycobacterial control but excessive production drives pathological neuroinflammation. TNF-α promotes granuloma formation yet increases BBB permeability and meningeal inflammation [11,31], providing a rationale for TNF-targeted therapies. IFN-γ activates macrophages, but elevated CSF levels are associated with poor outcomes, including stroke: meta-analyses demonstrate elevated CSF IFN-γ in TBM [32], and higher CSF IFN-γ, TNF-α, and IL-6 were observed in those who developed stroke in a cohort of 129 TBM patients [33*]. Although CSF IL-1β is elevated in TBM patients compared with controls, it does not predict mortality or disease severity, suggesting that IL-1-targeted strategies may have limited impact [32, 34].

#### Tryptophan

**Figure 1:**
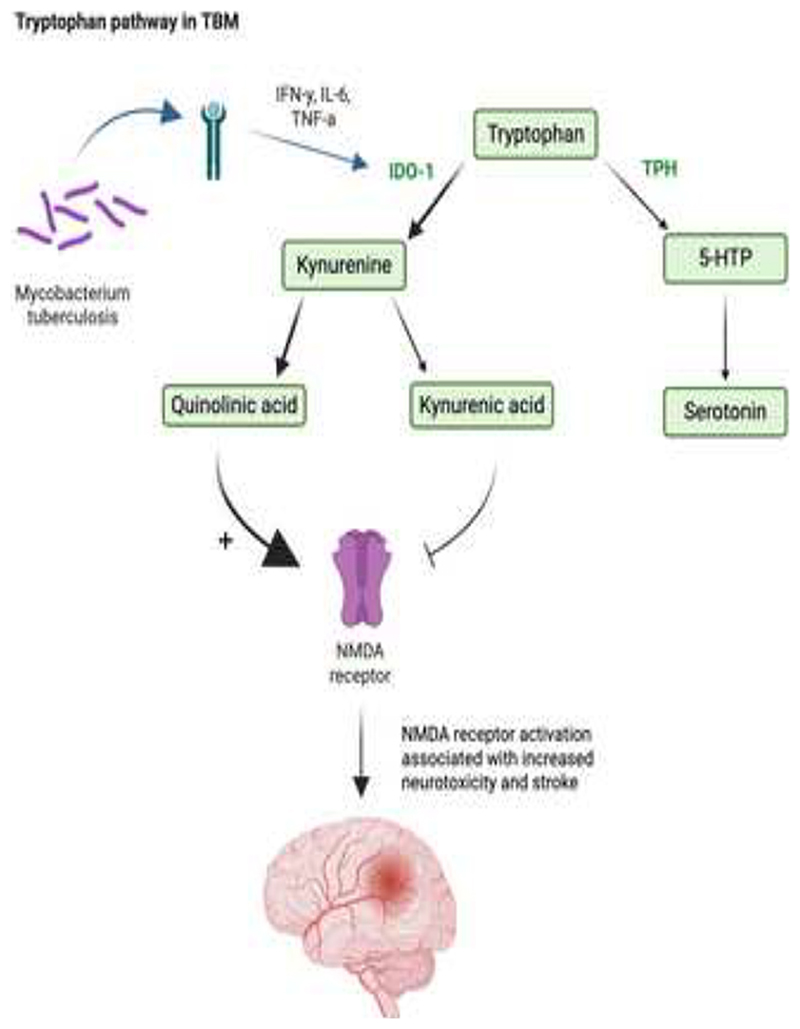
Tryptophan pathway in Tuberculous Meningitis. Tryptophan, an essential amino acid, can be metabolised by both the kynurenine and serotonin pathways. Pro-inflammatory cytokines released in response to *Mycobacterium tuberculosis* infection stimulate IDO-1, and preferential activation of the kynurenine-tryptophan pathway. Accumulation of quinolinic acid results in increased NMDA receptor activation, associated excitotoxic neuronal injury and stroke. Abbreviations: IFN-γ – interferon gamma; IL-6 – interleukin-6; TNF-α – tumour necrosis factor alpha; IDO-1 - indoleamine 2,3-dioxygenase; TPH – tryptophan hydroxylase; 5-HTP – 5-hydroxytryptophan Created in BioRender. Lamb, G. (2026) https://BioRender.com/ref9bxc

Tryptophan metabolism via the kynurenine pathway has been implicated in TBM pathogenesis (Figure 1). IFN-γ, IL-1β, IL-6 and TNF-α upregulate indoleamine 2,3-dioxygenase (IDO1), shunting tryptophan into the kynurenine pathway and generating neuroactive metabolites including quinolinic acid, an NMDA receptor agonist implicated in excitotoxic neuronal injury and stroke [11, 35].

In patients with TBM, higher baseline CSF tryptophan level is associated with increased mortality [36, 37*]. In >1000 TBM patients, CSF tryptophan independently predicted death and was inversely associated with IFN-γ, a key inducer of IDO1, whilst downstream metabolites of the kynurenine pathway correlated with CSF inflammation but not mortality [37*]. Plaatjie et al. showed greater CSF quinolinic acid accumulation in TBM compared to other CNS infections, with reduced CSF 5-hydroxytryptophan (5-HTP) indicating preferential activation of the tryptophan-kynurenine pathway [38**]. Quinolinic acid was associated with basal meningeal enhancement and hydrocephalus on MRI – key predictors of stroke [38**, 39]. Tryptophan-kynurenine pathway metabolites are linked with neuro-inflammation, supporting investigation of IDO1 inhibition, but additional unrecognised factors mediate the link between tryptophan and mortality.

Broader metabolic dysregulation may contribute to TBM pathology. Thuong et al confirmed that higher baseline CSF tryptophan levels predicted mortality in TBM, but found a stronger association between mortality and increased CSF 3-hydroxyoctanoate, part of a cluster of hydroxylated fatty acids that correlated with inflammatory markers [40*]. Whether altered tryptophan or fatty-acid metabolism directly influences stroke risk warrants further study.

### Part 2: Clinical and radiological predictors of infarction in TBM

Early identification of patients at high stroke risk could aid prognostication and guide targeted host-directed therapies. Previous studies have linked radiological markers of inflammation (basal exudates, meningeal enhancement), and inflammatory biomarkers (raised C-reactive protein, elevated CSF protein and low CSF glucose) with stroke in TBM, supporting a relationship between inflammation, vasculopathy and ischaemia [39, 41*].

In one of the largest studies to date, Guo et al. reported a 20.4% incidence of new stroke within 30 days of ATT initiation among 1,353 TBM patients [42**]. Independent predictors of stroke included age >35 years (OR 1.49), hypertension (OR 3.56), diabetes (OR 1.78), smoking (OR 2.88), TBM severity (OR 2.11 for MRC grade ≥II), meningeal enhancement (OR 1.66) and hydrocephalus (OR 2.98). Although stroke strongly predicted 90-day disability, it mediated less than 20% of the association between neuroimaging markers of inflammation and disability, suggesting additional mechanisms such as diffuse cerebral ischaemia and hypoperfusion without overt stroke.

In contrast, Aggrohia *et al*. found no association between basal exudates or meningeal enhancement and infarction in a younger cohort with few vascular risk factors [43**]. In this study, 43% of patients had MRI-confirmed infarction, yet vasculitis was detected in fewer than half of those undergoing magnetic resonance angiography (MRA), suggesting thrombosis in absence of vessel wall inflammation visible on MRA. Infarction was most strongly associated with hydrocephalus, hypertension at presentation, reduced Glasgow Coma Scale (GCS) score and shorter symptom duration. Hydrocephalus likely promotes ischaemia through raised intracranial pressure (ICP) resulting in impaired cerebral perfusion. Vomiting, a clinical marker of raised ICP, independently predicts stroke in TBM [41*] as does systemic hypertension, which may reflect compensatory attempts to maintain cerebral perfusion [42**].

Subgroup analysis from Guo et al. showed that conventional vascular risk factors potentiated the effect of meningeal enhancement, but not hydrocephalus, on stroke risk [42**]. Patients with pre-existing endovascular dysfunction may be more susceptible to TB-related vasculitis and thrombosis, whereas raised ICP may predominate in those without vascular comorbidity. This is supported by paediatric data: children rarely have vascular risk factors, yet experience higher rates of hydrocephalus, infarction, and neurological sequelae exceeding 50% [44]. Among TBM patients without vascular comorbidity, disease severity remains the strongest predictor of stroke, although inflammatory biomarkers also retain prognostic value [45].

**Figure 2:**
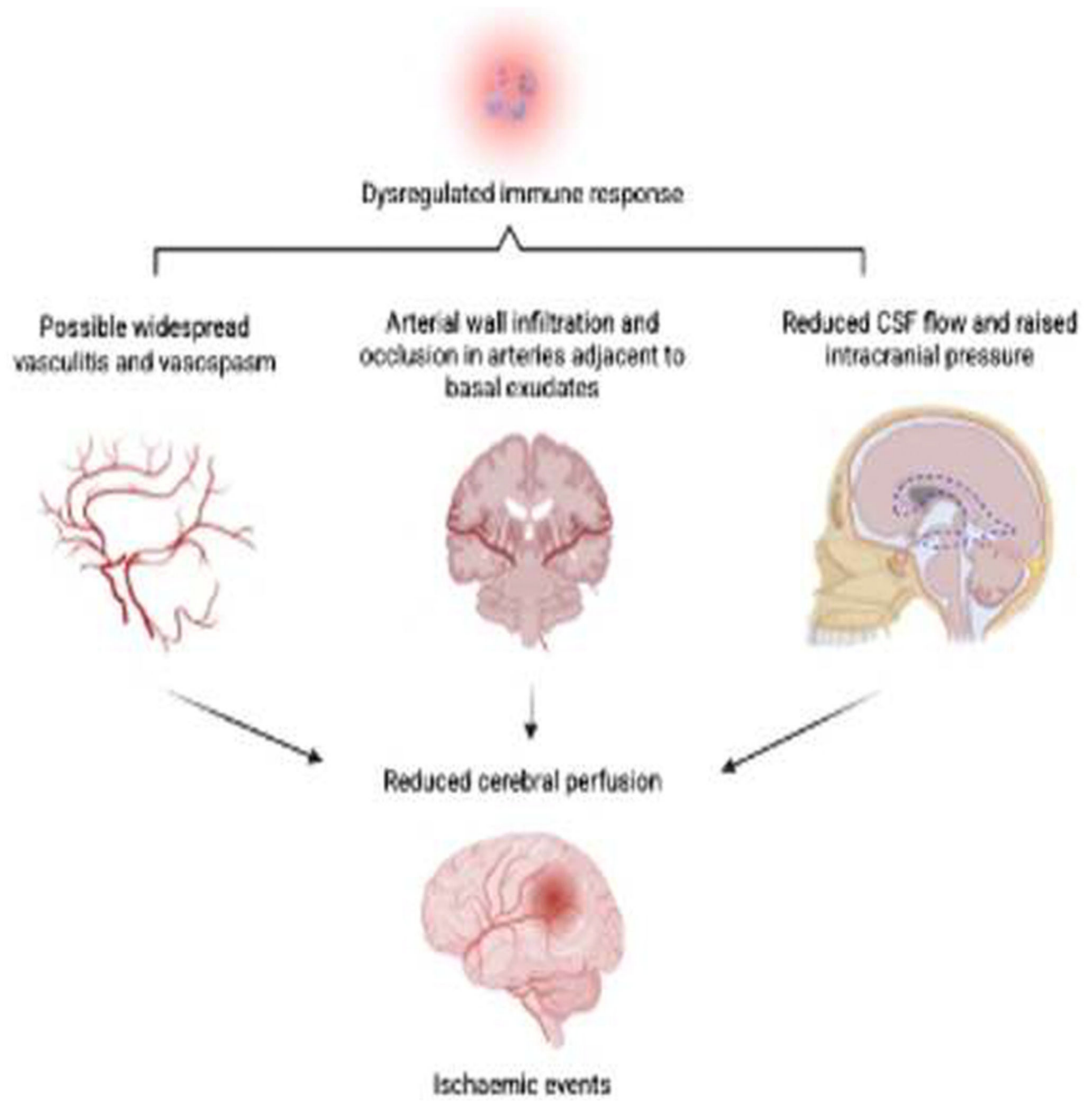
Schematic to describe contributing processes in the development of ischaemia in TBM. The dysregulated immune response leads to inflammatory exudate in the basal cisterns which causes direct arterial wall inflammatory changes in vessels in close proximity to the inflamed meninges. Inflammatory vasculopathy and vasospasm occurs more widely leading to reduced vessel calibre in small arteries of the brain. Simultaneously reduced CSF flow, vasogenic and cytotoxic oedema lead to raised pressures within the CNS. These processes contribute to reduced cerebral blood flow and subsequent ischemic changes in the brain.

Collectively, these findings suggest that both neuroinflammation and raised ICP contribute to TBM-associated ischaemia, with their relative importance varying by age and comorbidity (Figure 2). Non-invasive ICP monitoring strategies, such as blood pressure and optic nerve sheath diameter measurement, may offer affordable tools for early risk stratification [46]. Future research should clarify the temporal interaction between inflammation and ICP, how this impacts on stroke risk, and determine whether more aggressive management of hydrocephalus reduces stroke risk.

### Part 3: Advances in radiological characterisation of CNS ischaemia and infarction

Cerebral infarction in TBM was historically believed to occur predominantly within the ‘tubercular zone’, comprising the head of the caudate nucleus, internal capsule, and anteromedial thalamus, attributed to basal exudates and arteritis of vessels at the base of the brain. [47]. However, contemporary MRI-based studies, which are more sensitive than CT for detecting meningeal enhancement and stroke, challenge this paradigm [48]. In a cohort of 90 TBM patients, although 57.7% patients developed infarcts on MRI, only 13% occurred in the ‘tubercular zone’ [49]. Subsequent studies have demonstrated frequent involvement of cortical and cerebellar regions, with the middle cerebral artery (MCA) and its perforators most commonly affected [43**, 50]. Perforating and terminal cortical branches may be particularly vulnerable to raised ICP, reinforcing the link between, basal meningitis, hydrocephalus and ischaemia. In paediatric TBM, MRI-detected infarction rates exceed those in adults, with the cerebral hemispheres (59%), basal ganglia (53%) and thalamus (26%) most frequently involved [24*]. An autopsy study of 51 TBM patients identified macroscopic infarction in 72%, significantly higher than clinically or radiologically detected infarction, predominantly in the MCA territory supporting classification of TBM-related infarcts by vascular territory rather than zonal anatomy [51]. Microscopic infarction and necrotising injury of terminal arteries were nearly universal, consistent with diffuse inflammation-driven ischaemia.

Advances in vessel wall imaging (VWI) have enhanced detection of cerebral vasculitis in TBM. Arterial wall enhancement correlates with infarction in corresponding territories and may precede luminal changes detectable on MRA [52]. Choudhary et al. identified infarction in 48.5% of 101 adults with TBM, while vessel wall enhancement was seen in 66.3%; vessel involvement on VWI was associated with disease severity [53]. In a paediatric cohort, MRA-proven arteriopathy was detected in 65% of children with TBM-associated stroke, and was associated with recurrent stroke, cortical involvement and mortality, supporting a role for arterial inflammation in ongoing ischaemic injury while on ATT [54*].

Machine learning approaches may further refine imaging-based risk prediction in TBM [8]. Canas et al. developed a prognostic model, integrating imaging and clinical data to predict disease progression and outcomes, demonstrating better accuracy in HIV-negative patients and severe disease [55*]. Similar models could be adapted to stratify stroke risk at diagnosis, enabling earlier identification of patients most likely to benefit from targeted host-directed or ICP-focused interventions.

### Part 4: Treatments targeting ischaemia in TBM

Given the immune-mediated nature of TBM pathology, host-directed therapies have been explored as adjuncts to ATT to reduce inflammation-driven thrombosis and ischaemia.

#### Antiplatelet therapy

Aspirin has been proposed as adjunctive treatment due to its antithrombotic and anti-inflammatory effects through irreversible inhibition of cyclo-oxygenase pathways and prostanoid production [56]. A 2021 meta-analysis suggested a reduction in new-onset stroke (hazard ratio 0.51; number needed to treat 10) but no clear mortality benefit with adjunctive aspirin [57]. Additional studies have since reported mixed findings (Table 1).

The 2025 ACT-TBM trial evaluated aspirin and clopidogrel for prevention of clinical stroke, imaging-confirmed infarction and mortality and found neither agent significantly reduced stroke or death compared with placebo [58*]. High baseline disease severity and a substantial prevalence of stroke at enrolment may have limited the ability to demonstrate benefit, reflecting the late presentation typical of TBM in routine practice.

Low-dose aspirin (75mg daily) was used in ACT-TBM, but higher doses (>150mg) are required for anti-inflammatory effects. Earlier high-dose trials were inconclusive: Mai et al. reported reduced infarction and death with 1000mg daily [59], whereas the LASER-TBM trial showed no mortality advantage of high dose aspirin in HIV-associated TBM (although underpowered for efficacy endpoints) [60*]. Importantly, no trial has demonstrated significant bleeding risk with adjunctive aspirin [57–60]. The 2025 SURE trial in children with TBM found no benefit of high-dose aspirin, with a non-significant trend toward increased harm (22% vs 15% combined death/severe disability in aspirin vs placebo groups) [61*]. The ongoing phase 3 ITENSE-TBM trial in sub-Saharan Africa is assessing whether intensified ATT or aspirin 200 mg daily reduces mortality [62].

#### Corticosteroids

To date, corticosteroids are the only host-directed therapy shown to reduce mortality in TBM, but they have little effect on stroke incidence or disability [10, 63]. Despite two decades of routine use, there is little evidence steroids prevent cerebral ischaemia, and their benefit appears largely unrelated to vascular complications [9].

#### Anti-TNF therapies

TNF-α has emerged as a key cytokine associated with neuroinflammation and infarction, prompting interest in anti-TNF strategies.

##### Thalidomide

Thalidomide was the first agent with anti-TNF activity studied in TBM after animal models demonstrated reduced neuroinflammation and improved survival [64]. Systematic reviews suggest potential benefit in paradoxical reactions, and that low doses (3-5mg/kg/day) appear relatively safe [65]. A 2025 case series of four patients with HIV-associated TBM-IRIS (two with hydrocephalus, one with infarcts) reported rapid clinical and radiological improvement with thalidomide [66*]. Evidence remains limited to small series, and routine use cannot be recommended.

##### Infliximab

Off-label use of infliximab has been described in refractory TBM and TBM-IRIS. A retrospective Indian cohort reported improved disability-free survival in severe TBM, including patients with infarction, with infliximab compared with matched controls managed with ATT and steroids alone [67**]. A subsequent case series of 18 TBM patients treated with infliximab following clinical deterioration showed encouraging outcomes: at initiation, one-third had infarcts and one-third vasculitis, with a median modified Rankin Scale (mRS) score of 3.5 [68**]. After one month, 38% improved by ≥1 mRS point and 71% had radiological improvement; at one year, survival was 94% and disability-free survival 71%. While promising, these uncontrolled data require confirmation in prospective randomised controlled trials.

#### Other experimental treatments

Isolated reports describe successful use of IL-1 inhibitor anakinra for TBM paradoxical reactions, though without specific evaluation of stroke [69]. Recognition of MMPs in TBM pathogenesis has prompted interest in MMP inhibition as a therapeutic strategy [70]; the phase 2 DIRECT trial is underway to determine whether adjunctive doxycycline, an MMP inhibitor, reduces mortality or severe neurological deficit in TBM [71].

## Conclusion

Cerebral ischaemia and infarction in TBM arise from a complex interaction between neuroinflammation, vascular injury and raised ICP. Despite decades of research, current adjunctive therapies provide limited protection against infarction, underscoring the need for earlier identification of high-risk patients and mechanism-guided intervention. Future research should prioritise integrated approaches that examine the link between inflammation, immunothrombosis, and raised ICP, harnessing advanced neuro-imaging and systems immunology approaches, alongside well-designed clinical trials evaluating host-directed strategies. In parallel, efforts should focus on optimising existing management strategies, particularly defining the most effective management of raised ICP.

## Key points

- Understanding of the pathophysiology of inflammation in TBM aids our understanding of ischaemia, which is linked to several key inflammatory pathways including platelet and neutrophil activation and excess of pro-inflammatory cytokines TNF-α, IFN-γ and IL-6.
- NETs and MMPs may play a key role in pathogenesis of stroke in TBM and the role of MMP inhibitors as adjunctive therapy in TBM merits further research.
- Features of neuroinflammation on MRI (leptomeningeal enhancement and basal exudates), and hydrocephalus are independent predictors of stroke in TBM, and early use of MRA or VWI could help to predict those at greatest risk of stroke.
- Raised ICP likely drives ischaemia in TBM, particularly in those without underlying vascular risk factors and particularly in children; clinical studies to determine the optimum management of ICP in TBM are needed.
- Clinical trials to determine the benefit of adjuvant aspirin in reducing stroke are inconclusive, and suggest no mortality benefit. Results of ongoing large phase 3 trials are awaited, but other possible targets to reduce immunothrombosis in TBM should be considered.

## Figures and Tables

**Table 1:** Randomized and Planned Trials of Aspirin in Tuberculous Meningitis.

Trial	Population (N)	Sites	Aspirin dosing	Comparator	Key outcomes	Stroke-specific outcomes
Misra et al. 2010	Adults with TBM (n=118)	India - single centre	Aspirin 150mg/day for 3 months	Placebo + ATT	Significant reduction in 3-month mortality; aspirin well tolerated	MRI-proven stroke at 3 months: 24.2% aspirin vs 43.3% placebo; not statistically significant
Schoeman et al. 2011	Children with TBM (n=146)	South Africa - single centre	Low dose (Aspirin 75mg/day) vs high dose (Aspirin 100mg/kg/day) regimens	Placebo + ATT	No significant difference in mortality or neurological disability; acceptable safety	Not prespecified focused endpoint; no specific stroke outcome reported
Mai et al. 2018 (NCT02237365)	HIV-uninfected adults with TBM (n=120)	Vietnam – single centre	Aspirin 81mg/day or 1000mg/day for 60 days	Placebo + ATT	Aspirin safe with standard ATT + dexamethasone; trend toward better outcomes in microbiologically confirmed TBM subgroup	Composite: new brain infarction on MRI or death by day 60: Placebo 28.9%; Aspirin 81 mg 22.2%; Aspirin 1000 mg 15.8% (p=0.40 overall). Subgroup confirmed TBM: 34.4% events (placebo) vs 14.8% (81 mg) vs 10.7% (1000 mg) (p 0.06 trend toward benefit)
LASER-TBM trial, 2023 (NCT03927313)	HIV-positive adults with TBM (n=52)	South Africa - 4 hospitals	Aspirin 1000 mg/day (with intensified regimen)	SOC ± intensified ATT	Safety study; primary AESI/death outcomes; efficacy not demonstrated	No dedicated stroke endpoint reported; functional outcomes similar and no clear infarct data
ACT-TBM trial, 2025	Adults with TBM (n=237)	India – multi-center	Aspirin 75 mg/day	Standard ATT alone	No difference in mortality or functional outcome at 3 months	Clinical stroke + imaging infarction composite: At 1 month: 9.1% (aspirin) vs 14.3% (standard) — not statistically significant; at 3 months: 6.7% vs 5.1% — no benefit
ISURE trial, 2025	Children with TBM (n=369)	Asia – India, Vietnam; Africa – Uganda, Zambia, Zimbabwe	Aspirin 20 mg/kg daily for first 8 weeks	Placebo	No significant difference in mortality with nonsignificant trend towards harm (22% severe disability/ death in aspirin group vs 15% in placebo group)	No stroke-specific outcomes yet published
INTENSE-TBM (ongoing) (NCT04145258)	Adults and adolescents with TBM (planned n~768)	Africa - Côte d’Ivoire, Madagascar, South Africa, Uganda	Aspirin 200mg/day (first 8 weeks), alongside intensified ATT	Placebo	Not yet published	Stroke / cerebral infarction is included as a secondary outcome

Abbreviations: ATT – antituberculosis therapy; SOC – standard of care; MRI – magnetic resonance imaging; TBM – tuberculous meningitis.
